# A misdiagnosed case of osteoid osteoma of the talus: a case report and literature review

**DOI:** 10.1186/s12891-017-1413-8

**Published:** 2017-01-23

**Authors:** Huan He, Hailin Xu, Hao Lu, Yu Dang, Wei Huang, Qing Zhang

**Affiliations:** 1 Department of Orthopaedics, Jiangyou 903 Hospital, Mianyang No. 9 Huafengxincun, Jiangyou, Sichuan 621700 China; 2 Department of Trauma and Orthopaedics, People’s Hospital, Peking University, South Xizhimen Street No. 11, Beijing, Xicheng District 100044 China; 3 Medical Affairs Department of Hubei Province Traditional Chinese Medicine, Luoyu Road No. 856, Wuhan, 430074 China

**Keywords:** Osteoid osteoma of the talus, Computed tomography, Ankle arthritis, Ankle instability, Case report

## Abstract

**Background:**

Osteoid osteoma (OO) is a common benign bone tumour that is rarely found in the talus. Its nidus is difficult to detect on early imaging. The atypical symptoms of OO and the presence of concurrent trauma or sports injuries may lead to misdiagnosis and delayed treatment. We herein analyse a case of misdiagnosis of OO of the talus and discuss how to improve the early diagnosis of this rare lesion, thereby permitting rapid treatment.

**Case presentation:**

A 23-year-old man with a history of left ankle sprains and chronic pain was diagnosed with another ankle sprain and managed conservatively based on normal X-ray findings. After 1 year of recurring pain, he was diagnosed with ankle traumatic arthritis and underwent arthroscopic surgery. His preoperative ankle X-ray findings were still normal, and magnetic resonance imaging at that time demonstrated bone marrow oedema of the left talus. His symptoms reappeared shortly after surgery and progressively worsened. Magnetic resonance imaging performed 3 months after surgery demonstrated widespread bone marrow oedema of the talus. The patient presented to our hospital for pain assessment and was diagnosed with OO of the talus 3 years after his symptoms began. Preoperative computed tomography (CT) demonstrated a typical nidus of OO of the talus. After a second surgery, the patient’s symptoms completely resolved, and pathologic examination confirmed that the lesion was OO. The patient recovered 3 months later and was able to walk normally.

**Conclusions:**

OO of the ankle joint exhibits a progressive course and is difficult to diagnose at an early stage. Patients with OO of the talus often have atypical imaging findings, no signs of ankle instability, and no anterior talofibular ligament tenderness. CT is valuable for diagnosing OO, although multiple CT scans may be required to detect the nidus. Proper imaging helps doctors to achieve the correct diagnosis early in the disease course, significantly shortening the treatment cycle and improving the patient’s quality of life.

## Background

Osteoid osteoma (OO) is a benign bone tumour first reported by Jaffe [1] in 1935. It accounts for approximately 13.5% of benign bone tumours and 2% to 3% of all primary bone tumours [2, 3]. The tumour rarely exceeds 1.5 cm [4] and consists of a central area (nidus) that is surrounded by dense sclerotic bone [5]. This lesion is more frequently found in men (male:female ratio of 2:1) and is commonly diagnosed between 5 and 25 years of age [6]. It occurs in the metaphysis and diaphysis of long bones, particularly the femur and tibia. OO of the foot is unusual, accounting for approximately 2% to 10% of all cases, and is a rare cause of joint pain [7–9]. In total, 97% of cases of talar OO are found in the talar neck [10]. OO typically presents with local pain that is most severe at night and that can be relieved by nonsteroidal anti-inflammatory drugs (NSAIDs). Depending on the location of the OO, patients may present with local swelling and tenderness, bony deformity, gait disturbances, or muscle atrophy [5, 11]. OO can be managed conservatively. When the OO-induced pain is too severe or does not respond to medication, invasive treatment options need to be considered. Traditional treatment of OO includes open or arthroscopic surgical excision of the lesion. Computed tomography (CT)-guided radiofrequency ablation and CT-guided laser photocoagulation have also been successfully used [12–14]. Patients who have undergone surgery require 3 weeks of restricted weight-bearing, whereas patients treated with radiofrequency ablation do not require weight-bearing restrictions [15]. We herein report a case of misdiagnosis of talar OO in a patient with a history of sprains and long-term chronic ankle pain. This OO was misdiagnosed as ankle traumatic arthritis because no nidus was detected on imaging. We discuss the cause of this misdiagnosis and highlight ways for clinicians to achieve the correct diagnosis in the setting of early symptoms and no imaging evidence.

## Case presentation

**Fig. 1:**
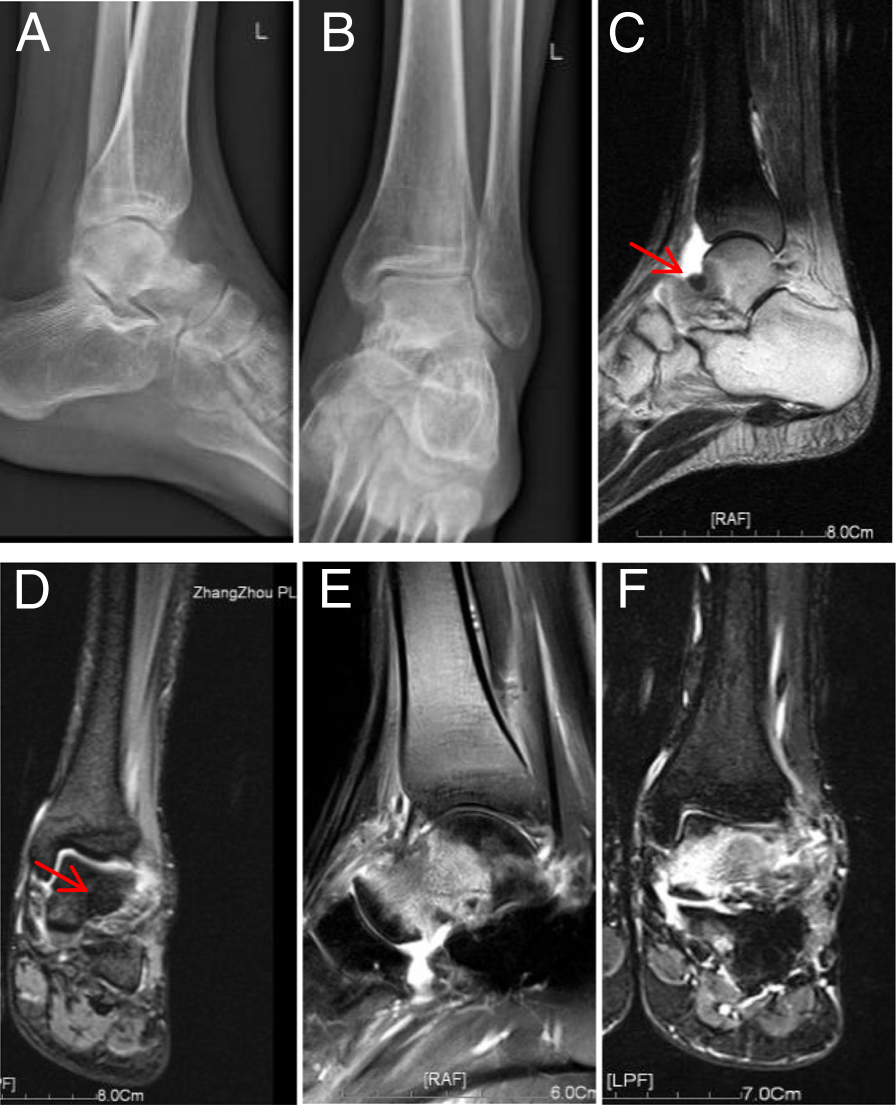
Preoperative and postoperative images at the time of the first surgery. (**a**,** b**) Preoperative lateral and anteroposterior X-rays show sclerotic lesions in the neck of the talus. (**c**, **d**) Preoperative sagittal T2-weighted and coronal T2-weighted magnetic resonance images of the left ankle show bone marrow oedema of the talus, left ankle joint effusion, and soft tissue swelling around the joint. An approximately 1-cm-diameter nodular shadow with an unclear boundary is seen in the upper front aspect of the left talus, and a small amount of effusion is present in the joint cavity (red arrow indicates the nidus). (**e**, **f**) Sagittal T2-weighted and coronal T2-weighted magnetic resonance images obtained 3 months after the first surgery show widespread bone marrow oedema of the talus

**Fig. 2:**
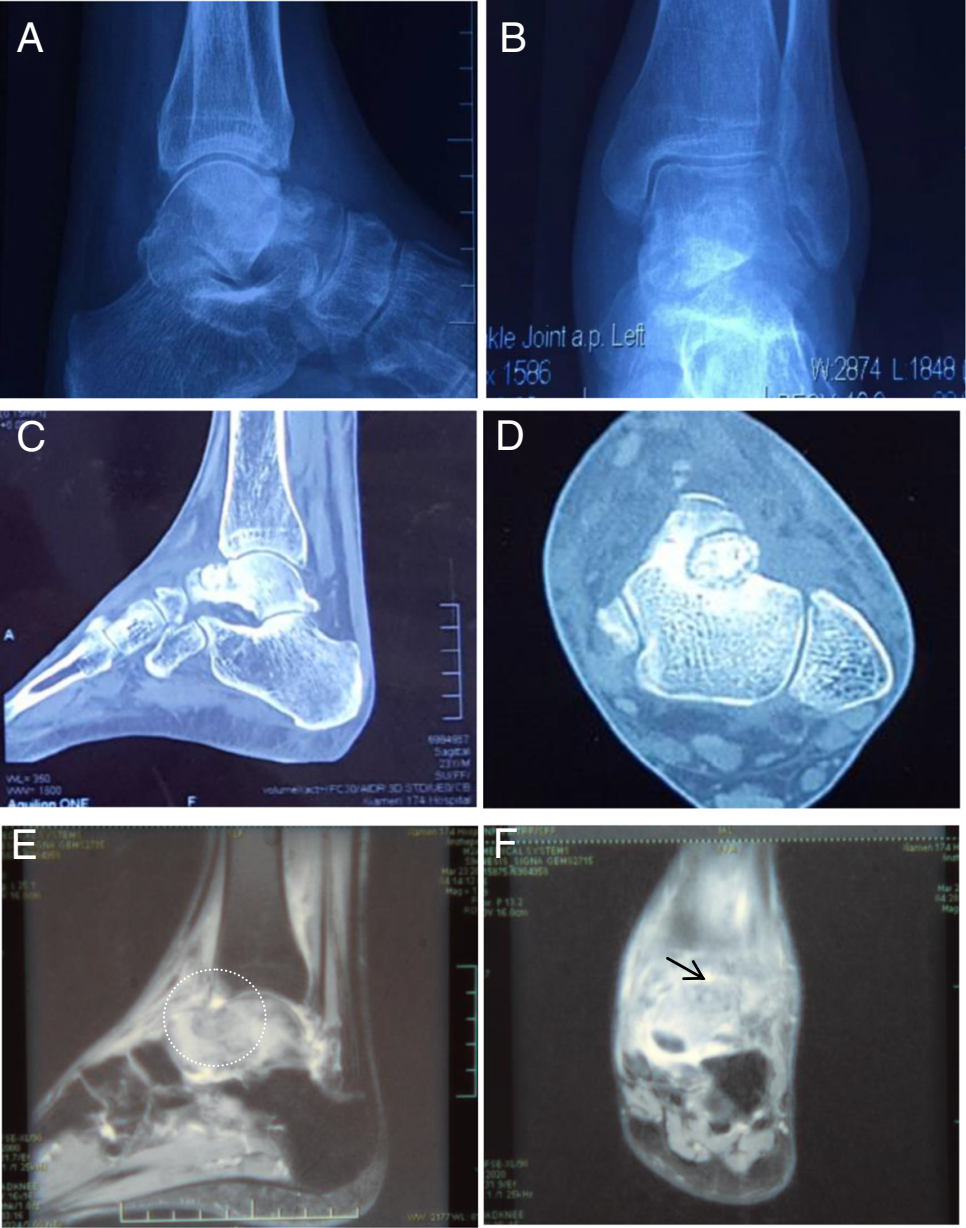
Preoperative imaging of the left ankle prior to the second surgery. (**a**, **b**) Preoperative radiographs show pathologic damage to the anterolateral talus, Approximately 1.0-cm-diameter higher-density nodules can be seen on the neck of the talus on the left side, with a clear boundary. (**c**, **d**) Preoperative computed tomography images show the characteristics of the osteoid osteoma nidus. (**e**, **f**) Preoperative sagittal T2-weighted and coronal T2-weighted magnetic resonance images show widespread bone marrow oedema of the talus with the osteoid osteoma lesion inside (white circle and black arrow indicate the nidus)

**Fig. 3:**
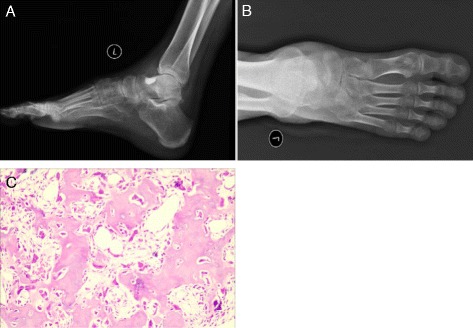
Postoperative imaging of the left ankle after the second surgery. (**a**, **b**) Postoperative radiographs of the left ankle show that the nidus had been completely removed. (**c**) Postoperative pathologic examination confirmed that the lesion was an osteoid osteoma (100×)

A 23-year-old man sprained his left ankle while playing football 3 years previously. He complained of left ankle swelling and chronic pain. His ankle X-rays were unremarkable. Following this initial presentation, his symptoms gradually improved, but he continued to note intermittent resting pain at night that was unrelated to activity. This night pain could become severe but was relieved by NSAIDs. One year after his injury, the patient presented for treatment because of recurring left ankle pain and swelling. X-rays showed sclerotic lesions in the neck of the talus (Fig. 1a, b). Magnetic resonance imaging (MRI) of his left ankle showed oedema in the talar bone marrow, left ankle joint effusion, and periarticular soft tissue swelling (Fig. 1c, d). He was diagnosed with ankle traumatic arthritis and underwent ankle arthroscopy at a local hospital. We referred to the patient’s record at the local hospital. The attending doctor performed arthroscopic debridement of the left ankle joint and found free small pieces of broken bone and osteophytes in the ankle joint during the surgery. However, no lesion was detected on the articular surface of the talus. We speculated that this may have been related to the limited arthroscopic field of view as well as the diagnostic level of the physician and his or her the lack of experience in arthroscopic techniques. The patient obtained short-term relief of pain for 3 months after the surgery because the arthroscopic debridement reduced inflammation around the joint. Additionally, long-term bed rest after surgery helped to temporarily control the symptoms. However, unless the nidus is completely removed in cases of OO, the symptoms gradually worsen after a short period of relief. The patient’s pain reappeared 3 months later, with more frequent night pain than before surgery. Postoperative MRI exhibited widespread oedema in the talar bone marrow (Fig. 1e, f). The attending doctor provided supportive treatment such as ice, compression, physical therapy, and oral NSAIDs to ease the pain. During the following 2 years, the patient’s pain continued but remained unassociated with activity. When the patient presented to our hospital, he could hardly stand or walk. His left ankle joint was swollen with a slightly increased local skin temperature. His foot was diffusely tender, especially at the anterolateral talus, without exacerbation upon palpation of the anterior talofibular ligament (ATFL). The ankle anterior drawer test was negative. A typical OO nidus was demonstrated in the talus on X-ray (Fig. 2a, b), CT (Fig. 2c, d), and MRI (Fig. 2e, f). The patient subsequently underwent nidus excision and artificial bone grafting (Fig. 3a, b). Postoperative pathologic examination confirmed that the excised tumour was a 1.5- × 1.5- × 1.0-cm OO (Fig. 3c). The patient’s pain and swelling disappeared after the treatment. He was able to walk normally when he presented for follow-up 3 months later.

## Discussion

Table 1 shows the anatomic locations of previously described OOs and their characteristics [16].

**Table 1:** Characteristics of osteoid osteomas at different anatomic locations

Morphological and anatomical location of Osteoid osteoma	Characteristics
Intracortical	Dense sclerosis around the nidus
Periosteal	Periosteal reaction
Spongiosal	Produces very little reactive bone
Subarticular reactions	Simulates arthritis as it produces synovial

In the present case, the OO was subarticular. This type of OO presents with ankle joint pain, swelling, and obvious joint effusion in a manner similar to arthrosynovitis. When patients present for treatment after trauma, OO of the talus is rarely considered in the setting of long-term ankle joint pain. Without a detailed history and physical examination, it is easy to miss OO or misdiagnose it as arthrosynovitis [17–19], ankle impingement [20–22], tubercular arthritis, or osteomyelitis [23]. This may lead to incorrect treatment at an early stage. A similar misdiagnosis may occur for OO of the knee joint [24].

The patient in the present case received treatment at his local hospital at the time of the first sprain and at the time of the arthroscopic surgery 1 year later. Although we were unable to obtain the X-ray images because the local hospital could not save them, a large amount of useful information can be obtained by analysing this case.

Obtaining the correct diagnosis at an early stage in the current case was very difficult because the early symptoms and imaging findings were both atypical [25]. The original physician did not fully consider the patient’s complaint of resting pain at night. When the arthroscopy was performed, the physician also did not notice that the patient’s ankle was stable without any obvious signs of laxity. These features are not consistent with traumatic arthritis secondary to ankle instability. The original physician ordered X-ray and MRI examinations without CT imaging. For OO of the talus, X-rays may be normal and the early-stage tumour nest may be obscured by bone marrow oedema and joint effusion on MRI. These factors could have led to the patient’s original misdiagnosis of arthrosynovitis [26, 27]. On closer examination, we noted unusual sclerotic lesions in the neck of the talus on the preoperative X-ray image taken at the time of the first arthroscopic surgery (Fig. 1a). However, the attending doctor noticed neither these lesions nor a tiny nidus on the MRI obtained prior to the first ankle arthroscopic surgery (Fig. 1c). When oedema of the talar bone marrow was noted instead of a nidus, the original physician did not consider the possibility of OO of the talus, resulting in the misdiagnosis.

Talar OO, although rarely detected, should be considered in patients with post-trauma chronic ankle pain that is atypical in nature, associated with episodes of resting pain at night, not correlated with activity, and relieved by NSAIDs [20] in the setting of a stable ankle joint without laxity, tears, pain on ATFL palpation, or imaging abnormalities. CT is considered the gold standard diagnostic technique for OO because it allows for accurate identification of the nidus [27], even when X-rays are negative [28]. Prior studies have confirmed that CT is superior to MRI in detection of the OO nidus [27–29]. Jordan et al. [30] performed an analysis of 223 cases of ankle OO. They found that the rate of missed diagnosis on MRI was 34% and that CT scans are the most valuable and accurate diagnostic imaging technique for these lesions. Pikoulas et al. [31] suggested that it is still necessary to use CT in cases of OO, even if the nidus is seen on X-ray. CT allows for accurate localisation of the nidus and assessment of the area around it, which assists with treatment decisions and surgical planning. If there is a high suspicion of OO, a CT scan must be performed [32]. CT is very important for the diagnosis of early-stage lesions. Radionuclide bone scans can also help to identify the tumour before the appearance of abnormal radiographic signs [26].

## Conclusion

In summary, OO in the ankle joint exhibits a progressive course and is difficult to diagnose, particularly during its early stage when characteristic symptoms are present but no lesion is detected. A patient with a history of chronic pain after trauma should be evaluated for OO of the talus and be advised to undergo regular re-examination. Patients whose illness history and imaging findings are atypical, do not display signs of joint instability, and have no ATFL tenderness should undergo CT scans to detect early-stage lesions. Multiple CT scans may be required to detect the nidus, but this imaging modality helps physicians to make the proper diagnosis as early as possible, thereby significantly shortening the lesion’s treatment cycle and improving the patient’s quality of life.

## Abbreviations

ATFL: Anterior talofibular ligament
CT: Computed tomography
MRI: Magnetic resonance imaging
NSAIDs: Nonsteroidal anti-inflammatory drugs
OO: Osteoid osteoma
